# Multiple senses of community and recovery processes. A pilot study for a national evaluation of the experiences of persons with substance use problems receiving help and services from Norwegian municipalities

**DOI:** 10.1002/jcop.22194

**Published:** 2019-04-30

**Authors:** Nina Kavita Heggen Bahl, Hilde Eileen Nafstad, Rolv Mikkel Blakar, Anne Signe Landheim, Morten Brodahl

**Affiliations:** ^1^ Department of Research and Development Clinic of Substance Use and Addiction Medicine, St. Olavs University Hospital Trondheim Norway; ^2^ Department of Psychology University of Oslo Oslo Norway; ^3^ Norwegian National Advisory Unit on Concurrent Substance Abuse and Mental Health Disorders Innlandet Hospital Trust Brumunddal Norway; ^4^ Norwegian Centre for Addiction Research University of Oslo Oslo Norway; ^5^ Innlandet University of Applied Sciences, Campus Elverum Elverum Norway; ^6^ Norwegian National Advisory Unit on Concurrent Substance Abuse and Mental Health Disorders Mental Health Division, Innlandet Hospital Trust Brumunddal Norway

**Keywords:** collaborate research, health services, interviews, qualitative analysis, recovery, Scandinavian welfare state (Norway), sense of community, substance use problems

## Abstract

**Aims:**

This pilot study uses a multifaceted concept of sense of community (SOC)—multiple senses of community (MPSOC)—to understand how the multiple communities of persons with substance use problems, including those with a positive, negative and neutral SOC, influence processes of substance use recovery.

**Methods:**

Semi‐structured interviews were conducted with 16 informants from different Norwegian municipalities and regions. A collaborative research design and thematic analyses with a peer researcher were applied.

**Results:**

The findings confirm prior findings of key ingredients related to recovery. However, they also illustrate that for communities to promote recovery, they need to fulfil individual needs, provide distance from pretreatment status, identity and roles and harmonise with individual meaning systems of an ideal community.

**Conclusion:**

Experiences of positive and negative community connections within geographical, relational and ideal communities take part in recovery processes. Community participation is suggested to be included in individual outpatient treatment and posttreatment plans.

## 1 INTRODUCTION

Humans have a deep need to belong and in our daily life, we are members of groups and communities such as family, working place, school and neighbourhood. Acknowledging then that in all stages of life, we are members of groups, communities and society (Bronfenbrenner, 1979; Nafstad, 2015) social memberships with its positive and negative consequences for development, health and well‐being have increasingly become a central research issue (Haslam, Jetten, Postmes, & Haslam, 2009; Jetten, Haslam, Cruwys, Haslam, & Steffens, 2017). To belong to or be part of groups and communities have also been shown to be among the key resources that promote initiation and maintenance of substance use recovery (Barbieri et al., 2016; Finney, Moos, & Mewborn, 1980; Kollath‐Cattano et al., 2018; Laudet, 2008; Moos, 2008; Pettersen et al., 2018; Schradle & Dougher, 1985). Adopting the perspective and concept of multiple senses of community (Brodsky & Marx, 2001; Brodsky, Loomis, & Marx, 2002) as our theoretical and analytical approach, the present pilot study's aims are to provide a more multifaceted and in‐depth understanding of personal experiences of positive and negative influences of multiple communities, in processes of recovery from problematic substance use.

## 2 SENSE OF COMMUNITY (SOC) AMONG PERSONS WITH SUBSTANCE USE PROBLEMS

Being members of different communities, people continually experience some form of bonding, of belonging or SOC. The feeling of being part of a community and the existence of care and social support are central for personal health, safety, subjective well‐being and social functioning (Ahern, Hendryx, & Siddharthan, 1996; Davidson & Cotter, 1991; Gattino, Piccoli, Fassio, & Rollero, 2013; Jorgensen, Jamieson, & Martin, 2010; Talò, Mannarini, & Rochira, 2014). Conceptually, the phenomena of being part of or SOC are understood as including four dimensions: (a) an experience of inclusion and identification with a community (membership); (b) different community and individual impacts (influence); (c) a sense that one's needs will be integrated and fulfilled through the community's resources (integration and fulfilment of needs) and (d) a more or less shared history of life and connections with other community members (shared emotional connection; Chavis, Lee, & Acosta, 2008; McMillan & Chavis, 1986). Moreover, our SOC can be of different types of affective experiences; we can experience a positive, neutral and negative SOC (Brodsky & Marx, 2001; Brodsky et al., 2002; Mannarini, Rochira, & Talo, 2014).

Studies have shown that people's SOC can play an important role in their substance use recovery (Barbieri et al., 2016; Drake, Wallach, & McGovern, 2005; Ferrari, Jason, Olson, Davis, & Alvarez, 2002; Jason, Davis, Ferrari, & Bishop, 2001; Kollath‐Cattano et al., 2018; Laudet, 2008; Peterson & Reid, 2003; Stevens, Jason, Ferrari, & Hunter, 2010; Stevens, Jason, Ferrari, Olson, & Legler, 2012). Both geographical communities (neighbourhood) and relational communities (school and sober environments) have been shown to moderate or prevent substance use and promote recovery for different groups of individuals with substance use problems (Battistich & Hom, 1997; Ferrari et al., 2002; Lardier, MacDonnell, Barrios, Garcia‐Reid, & Reid, 2017; Mayberry, Espelage, & Koenig, 2009; Stevens et al., 2010; Stevens et al., 2012). Most research on the influences of communities on substance use recovery has focused primarily on the role of sober living contexts or therapeutic communities (Oxford Housing; Stevens, Guerrero, Green, & Jason, 2018); and only one type of community (see Barbieri et al., 2016; Jason et al., 2001; Peterson & Reid, 2003). However, most people—here persons with substance use problems—belong to multiple communities: geographical (e.g., their hometown and neighbourhood) as well as relational (e.g., their family, friends and self‐help groups). Experiences in different communities provide alternative sources of pursuing life without problematic substance use. Moreover, through life people develop personal concepts or meaning systems of ideal living (Bahl, Nafstad, Blakar, & Geirdal, 2017; Glynn, 1981; Heintzelman & King, 2014; Resnick & Leddy, 2015; Sagy, Eriksson, & Braun Lewensohn, 2015) and meaning systems form and shape our feelings, decisions and behaviour. Having ideas or concrete meaning systems about ideal living, social support and community relations may motivate and improve recovery, as meaning systems are deeply subjective and thereby very influential.

## 3 MULTIPLE SENSES OF COMMUNITY AND PROCESSES OF RECOVERY

There are many definitions of recovery, but there is an agreement within substance use research that recovery has to be understood as a process for a better life, where the aim is to “recover” oneself as a person and be able to find meaning in a life without problematic substance use (Brekke, Lien, Davidson, & Biong, 2017; Kaskutas et al., 2014; Landheim, Wiik, Brendbekken, Brodahl, & Biong, 2016; Laudet, 2007; Miller, 2016). Most important, this pursuit for a better life necessarily takes place in contexts beyond the professional health care system (Brekke et al., 2017; Bronfenbrenner, 1994; Davidson & White, 2007; Landheim et al., 2016). It becomes life itself and entails everyday participation and meaningful activities in communities at a group as well as a societal level.

Thus, a variety of supportive community connections are likely to be important in the recovery process; in initiating a supported recovery process, in laps and relapse, in regaining health and the meaningful community memberships peoples had before their problematic substance use. People's SOC can thus shape and strengthen a hopeful and collaborative process for a meaningful life without problematic substance use (Barbieri et al., 2016; Brekke et al., 2017; Ferrari et al., 2002; Lardier Jr et al., 2017; Peterson & Reid, 2003; Stevens et al., 2010; Stevens et al., 2012). However, prior research on SOC among persons with substance use problems has assumed that SOC has to be of an outright positive experience with positive outcomes. To get a more nuanced and complete understanding of the roles SOC play in processes of recovery from problematic substance use, there is a need for research based on a multifaceted concept of SOC, acknowledging the different senses of community persons with substance use problems can experience in their multiple communities throughout their process of recovery.

Taking as our frame of reference that individuals with substance use problems are members of multiple communities where they, throughout processes of recovery, can experience positive, neutral or negative SOC, this study aims to explore and understand the different ways persons with substance use problems experience their communities and senses of communities as influencing processes of recovery from problematic substance use in a positive and negative manner. Moreover, inspired by the utilities of including community members, peer counsellors and peer support workers within substance use research—increased quality, relevance and validity of the study's findings (Davidson, Bellamy, Flanagan, Guy, & O'Connell, 2017; Gordon, Franklin, & Eltringham, 2018; Minogue, Boness, Brown, & Girdlestone, 2005)—we have chosen to use a collaborative research design to increase the understanding of interview data from informants with substance use problems living and participating in different communities in several Norwegian municipalities.

## 4 METHODOLOGY

### 4.1 Collaborative research design

To do research *with* rather than *on* persons recovering from substance use problems is not commonly undertaken, but has become a considered ideal inspired from mental health research (Faulkner, 2004; Nowotny, Scott, Gibbons, & Scott, 2001; Trivedi & Wykes, 2002). The perspective of individuals with experience of recovering from substance use problems was included in the present study through collaboration in all stages of the research. A peer support worker from the Drug and Alcohol Competence Centre in Central Norway participated in the reference group (resembling community advisory groups but restricted to peer support workers, researchers and clinicians as members) planning the project, data collection and developing the interview guide; all 16 informants interviewed had experiences with recovering from substance use problems; and a second peer support worker having recovered from substance use as well as with education and experiences with qualitative research collaborated with the first author as a peer researcher in analysing the material.

This peer researcher collaborated with the first author in coding the material and interpreting findings. Moreover, in a few cases, we have chosen not only to include the peer researcher as a coresearcher in the analysis, but we have also separately presented some of his interpretations of the informants experiences and contexts from his perspective and horizon based on his unique experiences of having himself had substance use problems in earlier life. This extra dimension of involving the peer researcher in the study was important as it broadened and strengthened the understanding of the findings.

### 4.2 Approach to enquiry

We have taken care to meet APA's standards for qualitative research (Levitt et al., 2018). As outlined above, the conceptual framework for the study has been developed deductively, whereas the collection of data was initiated by a more inductive thematic analysis (Braun and Clarke (2012). In relation to Braun and Clarke's (2012) three dimensions, the study is experiential in its orientation and essentialist and constructionist in its theoretical framework. The study is experiential and essentialist as the initial analysis of the material was inductively driven by what was in the data; by assuming a knowable world, and “giving voice” to the experiences of a particular group of individuals. The study is also constructionist, as the second deductive analysis moves beyond describing the social world of a group and examine how their social world is put together; the way multiple communities constructs the process of recovery from substance use problems.

### 4.3 Context: Services for persons with substance use problems in Norway

Individuals from different cultural and national contexts have collaborated in studies of SOC and recovery from substance use. Most of these studies are from North America (Curtis, Jason, Olson, & Ferrari, 2005; d'Arlach, Olson, Jason, & Ferrari, 2006; Ferrari et al., 2002; Jason et al., 2001; Lardier et al., 2017; Peterson & Reid, 2003; Stevens et al., 2010; Stevens et al., 2012), however, the present study is from a European context, a Scandinavian welfare state, Norway. In the Norwegian professional health care system, persons with substance use problems are offered services both from the regional state level (specialist health services) and locally from the municipal level. The Norwegian social, psychosocial and medical rehabilitation services are intended to help service receivers with their life and participation in society based on their own premises and goals.

### 4.4 Material

Our data are from a verbatim‐transcribed interview material was collected for a larger national evaluation (see Helsedirektoratet, 2018a, 2018b). This project was undertaken on assignment from the Norwegian Directorate of Health and Social Affairs to the drug and alcohol competence centre in central Norway to get to know the concrete experiences of the services from Norwegian municipalities for persons with substance use problems.

### 4.5 Recruitment and sample

A purposeful sampling strategy was used to recruit informants who had experience with services from Norwegian municipalities for individuals with substance use problems. Contact was established by email directly to leaders of services from different municipalities in different parts of Norway. The e‐mail included a formal invitation describing the purpose of the study and the inclusion criteria for informants; (a) having had contact with services from the municipality for a minimum of 3 months, (b) being over 18 years old and (c) to have an on‐going or ended substance use problem (including alcohol). The invitation also requested the leaders to ask employees if they knew any users of their services who would be interested to participate in an interview.

Four leaders from municipalities of different sizes with respect to residents (small, population below 5,000; medium, population below 20,000 and large, population over 20,000) agreed to participate in recruiting informants for the project. In the two large municipalities 11 informants were recruited from three different services. In the medium size municipality three informants were recruited from one service and, finally, in the small municipality two informants were recruited from one service.

In total, a sample of 16 informants (nine men and seven women) participated in this study. The samples age ranged from 24 to 77 years, with half of the sample being between 40 and 50 years old. With respect to recovery processes, the sample included informants that had different experiences and different length of recovery processes (see Table 1).

**Table 1:** Informant's background

Informant	Age	Size of municipality	Municipality services	Years of service utilisation	Relational communities (positive, + and negative, −)	Geographical communities (positive, + and negative, −)	Ideal communities
W24	24	Large	Integration and work assisting service, general practitioner, drug and addiction services	3	+: Integration service and work assisting service		
M29	29	Small	Drug and addiction services, work assessment allowance, general practitioner, interdisciplinary coordinating group	14	+ Interdisciplinary coordinating group	+: Cohabitation	
W37	37	Large	Concurrent substance abuse and mental health disorder‐team, addiction consultant, home nursing care	10	+: Family, concurrent substance abuse and mental health disorder service, religious community	−: Local community	Halfway‐houses
					−: Service related community		
W38	38	Large	Medically assisted delivery of opiates, housing assistance	23	+: Family		
					−: Family		
W42	42	Medium	Medically assisted delivery of opiates, home nursing care, drug and addiction services, addiction consultant, general practitioner, interdisciplinary coordinating group, housing assistance, physical exercise group	15	+: Interdisciplinary, coordinating group, exercise group, low‐threshold service		Substance‐free relations, creative communities
					−: Low‐threshold service		
M42	42	Large	Medically assisted delivery of opiates, general practitioner, day treatment centre	29			A place to do meaningful activities
M43	43	Large	Medically assisted delivery of opiates, social benefits	23	+: Medically assisted rehabilitation		
					−: Activity group offer from the municipality		
M43	43	Medium	Medically assisted delivery of opiates, general practitioner, make‐work programme	6	+: Make‐work programme		
W45	45	Large	Medically assisted delivery of opiates, general practitioner, addiction consultant interdisciplinary coordinating group	5	+: Family, addiction consultant, after care service	+: Local community	
					−: Family		
W46	46	Large	Aftercare programme, addiction consultant, general practitioner, interdisciplinary coordinating group	10	Interdisciplinary coordinating group		
W48	48	Large	Medically assisted delivery of opiates, general practitioner, housing assistance	13			Creative communities
W49	49	Large	Concurrent substance abuse and mental health disorder service, addiction consultant, day treatment centre, housing assistance, general practitioner	19	+: After care service, close friends, religious community (Christian), Day care centre, self‐help group,		
					−: AA		
M52	52	Medium	Addiction consultant, drug and addiction services, general practitioner, make‐work programme	4	+: Family, AA	+: Neighbourhood	A safe place to live
						−: Earlier housing by the municipality	
M53	53	Large	Medically assisted delivery of opiates, nurse in opioid maintenance treatment, general practitioner, Norwegian Labour and Welfare Administration, drug and addiction services	15		−: Shelter	
M53	53	Small	Drug and addiction services,	18	+: Family	+: Neighbourhood	
			Norwegian Labour and Welfare Administration, general practitioner				
M77	77	Large	Concurrent substance abuse and mental health disorder service, social worker, general practitioner	>25	+: Religious community (Christian), Senior Centre	+: Local community, Senior Centre	

### 4.6 Data collection

Semi‐structured in‐depth interviews were chosen to ensure that the informants could express their experiences without restrictions and at the same time give some structure to topics addressed in the interview. To use the same type of semi‐structured interview was also important as the larger national project planned follow‐up studies applying the same instrument in future follow‐up studies. The order of questions, however, was continually adapted with respect to the discourse of the informant in the interview. As a medium‐large qualitative research project, 16 informants were regarded as appropriate for the current interview study (see Braun & Clarke, 2015). All interviews were conducted by a clinical research assistant who had long experience both in collecting data as well as with individuals with substance use problems. Every interview was conducted in the location of the service which the informant had the closest contact with, as chosen by the informants. The interviews lasted between 21 to 63 min and were conducted between November 2017 and February 2018.

### 4.7 Interview guide

Like several other interview studies exploring the role of communities in people's lives (e.g., Brodsky, 1996 and Brodsky, 2009) the aim of the present study was to explore different aspects of a particular group's everyday life. An open topic guide was used to interview informants about their background (age, years of residency in the municipality, housing situation, family, health and substance use) as well as about the services from the municipality they used in their recovery process. The services asked about were related to housing, work, education, economy, leisure activities, and other needs. The topic guide also included questions about family and significant others, and how the collaboration between services for persons with substance use issues was experienced.

### 4.8 Analyses

The thematic analysis in this study was two‐part; one preliminary inductive thematic analysis and one deductive analysis. The first author was greatly involved in transcribing the interview material (9 of 16 interviews) to familiarise with the data. Transcribing, repeatedly listening to the remaining interview recordings, and discussions about the interviews between the first author and the interviewer made up the first step in the analyses of the material (familiarizing with the material). In the preliminary analysis, the first author explored themes and subthemes in the material in an explorative way (see Helsedirektoratet, 2018b). Six overarching themes were identified in this analysis: (a) health; (b) experiences with health services; (c) family, community and networks; (d) overview, insight and participation received services; (e) transitions and (f) collaboration. “Family, community and networks” was the most salient theme.

As the informants addressed several communities as central in their recovery process, it was decided to use both a contemporary as well as a multifaceted theoretical conceptualisation of SOC (see Brodsky, 2009; Brodsky & Marx, 2001; Brodsky et al., 2002) to more systematically reanalyse the material. Therefore, a deductive in‐depth thematic analysis was conducted (Clarke et al., 2015). To generate initial codes the material was coded by the first author according to the following community concepts: geographical, relational and ideal communities. In the process of coding, it became evident that the informants addressed the various communities they were part of in positive and negative ways; it was therefore ensured to include these more affective dimensions in the coding. The codes were systematised and defined as hierarchal nodes in NVivo 11 with “Communities” as an overarching code (parent node) with five subcodes (child nodes); geographical communities; negative geographical communities, relational communities, negative relational communities and ideal communities. Then, the first author and the peer researcher went back to the material connected to the five codes to identify additional codes and themes individually. The peer researcher read all transcripts. After this process, the two researchers met to share and discuss their individual codes and themes. Through the first discussion of the material, the peer researcher confirmed the initial theoretical coding of the material. In addition, each of the two researcher's codes and themes were added, reviewed and validated. Through a second discussion, five themes were defined as final themes. Finally, all authors collaborated in the final step of the analysis (producing the report) by describing and interpreting all themes in light of relevant concepts and research.

### 4.9 Ethical considerations

The project was approved by a regional ethical committee as well as the internal data protection officer at St. Olavs Hospital in Trondheim, Norway. Before participating in the study all informants were informed about what participation would entail. They were also informed about who would conduct the interview, that the interview would be digitally recorded and made anonymous before being transcribed as verbatim text, and finally, that participants could withdraw their consent at any time. All informants signed a consent form before the interviews were conducted.

## 5 FINDINGS AND DISCUSSION

As presented, research has shown that communities can be important for the initiation and maintenance of recovery from problematic substance use (Barbieri et al., 2016; Finney et al., 1980; Kollath‐Cattano et al., 2018; Pettersen et al., 2018; Schradle & Dougher, 1985). Moreover, the sense of being part of a community is important for personal development, health, safety, subjective well‐being and social functioning (Ahern et al., 1996; Davidson & Cotter, 1991; Gattino et al., 2013; Jorgensen et al., 2010; Talò et al., 2014). We will now describe the ways informants from different municipalities in Norway experience their multiple communities as shaping and influencing their particular recovery process.

Through the collaborative thematic analysis, multiple communities were identified in the informants' descriptions of their process of recovery. Geographical, relational and ideal communities were identified as sources of different forms of SOC and as influencing their recovery process in various positive and negative ways. The typical geographical communities mentioned were the municipality as a local community, housing offered from the municipality and their neighbourhood communities. Relational communities frequently addressed were family, friends and sober posttreatment communities like activity and self‐help groups (AA/NA) as well as religious communities (Christian). Finally, ideal communities were generally described as meeting places where one could use ones skills to do meaningful, positive and creative activities together with others.

### 5.1 Theme 1: Positive geographical communities

Most of the informants described geographical communities like the local community, housing offered from the municipality and their neighbourhood as in one way or the other challenging their recovery process. However, some experiences of geographical communities were identified as positively influencing the recovery processes.

#### 5.1.1 The municipality as a local community

According to the peer researcher, important elements of geographical SOC such as experiencing a sense of involvement, being valued and having an experience that actions are taken by the local community (in this case to deal with substance use problems) are elements important for recovery from substance use problems.

#### 5.1.2 Housing from the municipality

Several types of housing offered from Norwegian municipalities have been recognised as rather problematic for persons in their recovery as they have residents who use substances and there is easy access to obtain substances (Brekke et al., 2017; Dyb & Holm, 2015). However, some forms of municipality housing may be particularly appropriate for specific groups of persons with substance use problems. For instance, one of the older adult informants described that living in an age‐appropriate housing (housing for elderly from the municipality) promoted respect for other residents' needs for peace and prevented his use of substances:

I^1^: “Is that an OK place to live?”

M77^2^: “It is very calm”.

I: “Is it municipal …?”

M77: “There are only older adults living in that community and… there are older adults around there you know living around me”.

I: “So, then there isn't a lot of noise in the weekends and…?”

M77: “No, it is quiet and calm. I have never brought or used (substances) in that place. Gee, there are only older adult people around me. Who aren't well either. I would never do so”.

#### 5.1.3 Neighbourhood communities

Some of the informants described their neighbourhood as a community providing them with a sense of inclusion, which was positive for the recovery process. This seemed to depend on the neighbours not knowing too much about the informants past:

W42: “I could sense it from the neighbours in the community, you know, eventually… that they have… there were some who were so nice when I moved in, and now… a couple of months ago after finding out about my background… they barely greet me”.

The peer researcher confirmed this finding and expressed his own experience of neighbourhood communities as important for the integration in society and distancing from a substance use related identity after treatment. Moreover, as others have found, housing and neighbourhood were described as positive for recovery if one had a stable economy. A stable economy is likely to depend on receiving economic support from services or family. As such, the promoting factor of neighbourhood relationships can be understood as a matter of social recovery capital (Best & Laudet, 2010; Kollath‐Cattano et al., 2018).

### 5.2 Theme 2: Negative geographical communities

Several of the informants experienced their geographical communities (e.g., the local community and housing offered by the municipality) as negatively affecting their recovery process. According to the peer researcher, this had to do with the fact that their geographical communities were often experienced as unsafe, providing a sense of unworthiness and a source of negative identity and negative community relations.

#### 5.2.1 The municipality as a local community

Some of the informants said that the municipality's reputation—as troubled by large substance use problems—promoted a substance use related identity. For instance, a female 37‐year‐old woman who experienced a “we” within her municipality, explained that the community was known to be at the “top of the list” when it came to number of people with substance use problems compared with the number of residents:

F37: “Yes. After all, we are so many drug addicts in this community compared to the number of residents. We are on the top of the list. And you can imagine what that does to others' perception of us…”

In this case, as strong positive geographical SOC is, contrary for most cases, likely to be challenging for the recovery from substance use. According to studies of negative SOC (Brodsky, 1996; Mannarini et al., 2014) people who actively distance themselves from surrounding environments that are dangerous, unhealthy and unfit are able to cope better with such unfavourable community settings. In this situation, where ones geographical community promotes a substance use related identity, a negative SOC is most likely to be protective and positive for recovery.

Other informants explained that they were exposed to illegal substances in their everyday life within the municipality. This was a big challenge for their recovery:

M48: “…there (in the local community) I met all the substance abusers… That was not beneficial for my recovery. You are offered pills… and then it's really hard to say ‘no'”.

As also emphasised by the peer researcher, this was particularly the case in areas of the municipality recognised as “ghettoes”.

#### 5.2.2 Housing from the municipality

In addition to geographical areas, there were special types of housings, which were described as making recovery especially challenging and most difficult. These included shelters and housing from the municipality which were regarded as places where recovery was hopeless as they had such a high degree of exposure to substances:

I: “Ok. Can you say a little more about what was messy about that (place to live)?”

M52: “It was the situation of living. I was placed in X (name of the location)”.

I: “When you came back from treatment?”

M52: “Yes. And there were alcohol and drugs everywhere”.

I: “And that was housing from the municipality, or was it a shelter?”

M52: “It was housing from the municipality. So, to recover from alcohol problems at that time, was something you could just forget about”.

This finding is consistent with other studies on housing from Norwegian municipalities (Brekke et al., 2017; Dyb & Holm, 2015), and just as with the larger local community, a negative SOC within housings offered from the municipality—to keep distance and limit ones presence in these unhealthy and unfit ways of living—is likely to be prohibited for individuals in recovery.

### 5.3 Theme 3: Positive relational communities

The active ingredients that facilitate recovery from substance use problems (e.g., social bonding, goal direction, modelling and building recovery skills) in different communities like family, friends and posttreatment communities (e.g., self‐help groups) are closely related (Moos, 2008). In fact, parallel groups (patients receiving help from mental health services) who have social support from such communities, also often experience the help from the professional help system to be more positive (Kogstad, Mönness, & Sörensen, 2013). Three relational communities were frequently addressed by the informants as having a positive influence on their recovery; family, friends and sober posttreatment communities.

#### 5.3.1 Family members

Family members contributed to the recovery process through their strong social bonds, help and support:

W37: “I'm thinking that what mom and dad did for me, was exactly what they should have done. They have fought for me. Yes, they didn't give up. Many of the parents of my friends have given them up. Their parents have just ended all contact with them. I have always had their support and help, sort of. So, thank God for that”.

Another way family was described to promote recovery was by making sure that the person in focus was assured to receive needed services and having some meaningful activities in their everyday life:

W24: “No, it is him (dad) who has fought the battle for me to get treatment”.

M53: “…it is my father who has bought that house which I have been renovating”.

I: “Yes, so that you have something to do? That's very clever”.

M53: “Yes, it works for me”.

Consistent with findings in other studies, this illustrates that those who have strong bonds with communities which can offer facilitate recovery (in this case family) are more likely to get in contact with other recovery facilitating communities (e.g., therapeutic communities and work; Kollath‐Cattano et al., 2018; Pettersen et al., 2018).

Finally, members from the family were also described by some as providing necessary stability and support after their treatment, needed to continue the process of recovery. For example, one informant mentioned the importance of having a stable place to live after treatment to prevent frequent contact with earlier substance users:

W38: “Yes. After treatment, they said that I had to retrieve (Subutex) every day because I didn't have a residence… but luckily I could live some days with my mother and then I could retrieve every other day. I am counting on that the next step in the process is that get my own apartment so I can retrieve even less frequent”.

As shown, for some the family was as an important community supporting recovery before, under and after treatment.

#### 5.3.2 Friends

Friends, particularly sober friends, were described by the informants as important for their recovery. Friends were often a source for contact with the needed services and offers:

M29: “Yes, I had a friend, in X (place), which had conversations with her (drug and addiction consultant). I think it was her (the friend) who put me in contact with her who have helped me so much”.

M53: “I learned about it (home loans) through a friend. He got it three months before me, and I received an answer about my application three months later, when I got it”.

In addition to family, new and sober friends were thus mentioned as important for a process of recovery after treatment:

I: “…but what would you have needed after treatment?”

M53: “… I think… I needed help to build a sober circle of friends, sort of”.

This finding confirms that the way family and friends facilitate recovery from substance use are closely related. More to the point, the findings point out that social recovery capital is not only facilitated by family but also by new and sober social relations such as friends. Friends can facilitate receiving service opportunities, as well as establishing new sober relations after treatment: both key components in recovery (Brekke et al., 2017; Kollath‐Cattano et al., 2018; Pettersen et al., 2018).

#### 5.3.3 Sober posttreatment communities

Sober posttreatment communities as after‐care groups, self‐help groups, activity and training groups, low‐threshold services, day‐care services, religious groups and make‐work programmes were described by the informants as promoting and supporting recovery. These communities are all systems of informal care. Mentioned elements of these communities important for facilitating the recovery process, included togetherness in the recovery process and meaningful and creative activities together. The most important component, however, was that these communities offered new and sober relationships and thus a detachment from a substance use related identity. As one of the informants who had completed treatment explained:

M53: “My community are people who I meet in the city everyday. When I go to my standard café there are so‐called straight people, who sit down and have a conversation with me. They don't know me, but they have seen me around. I think that's really nice”.

Other informants described these communities as promoting social competence development, fulfilment of needs and interests. Moreover, the informants described these posttreatment communities as a place for hope and sense of belongingness For instance, a 46‐year‐old female informant who participated in a self‐help group described:

W46: “That (self‐help group) has been a crucial community for me. There I got an explanation that I'm not just stupid, that I actually suffer from a condition which is called “addiction” and which drives me to do things contrary to my own interests and values, actually. It's a community where you belong and which helps you to stay sober”.

To have something meaningful to do, that being religious practice or creative activities, with others were additional key ingredients to how these communities promoted recovery:

W46: “You learn more about yourself and become a whole person through the community. And, there is spirituality also. I felt that when you just stop getting high, there is an empty space within you, and by using higher powers or a form of spirituality you don't become so alone and empty. So, it replaces substances, kind of”.

W49: “X (the activity centre) is an important community for me, because we are creative there. We can sow on the sowing machine, we can paint pictures, we can knit, we do things instead of thinking of substances, and then the days pass. You do things, you feel satisfied with what you do and then you come home and feel tired. Then everything is fine”.

Another way sober posttreatment communities contributed to the recovery process was by offering peer person relations and role models with longer experience of recovery. As one of the young informants pointed out:

I: “How has it been with regard to receiving help with establishing a network… do you think that X (after care) are involved in that?”

W24: “Yes, absolutely. Another thing with this place is that you meet people who have experience themselves… who have been sober for a longer time than you have, so you can pick up some advice with regard to that too. So X (after care) is absolutely a place for that, to build a community”.

I: “Yes, because here you have someone to reflect together with, or who can…”

W24: “Yes, or others who attend X (after care) and who have experiences themselves. Who may have done the mistake that they have worked too much and not been social enough. You meet people and learn from them”.

Several of the functions which the informants described (e.g., belongingness, finding meaning through spirituality or activities, the importance of role models and training social function) have also been shown as active ingredients in informal care systems which facilitate recovery from substance use problems (Moos, 2008). In fact, the way these communities are described confirm that these communities contain several elements conceptually overlapping with both SOC, recovery and recovery capital (Best & Laudet, 2010; Brekke et al., 2017; Moos, 2008; Stevens et al., 2018).

### 5.4 Theme 4: Negative relational communities

Although relational communities often are based on shared interests and common purposes with other members, relational communities which people have negative connections to, can be a source of negative SOC as well as being negative for the process of recovery. Some informants also described the family and sober posttreatment communities as sources of frustration (for not fulfilling ones needs) and abstention (refraining any type of action from the social domain), which are both components of negative SOC. Mannarini et al. (2014) have been particularly interested in these relational connections and negative SOC.

#### 5.4.1 Family members

There were several examples in the material of descriptions of family members as a source for rejection, conflict and risk to own recovery:

M29: “…my mother has been doing a deal of substances herself, and it's just her that I have had in my life. I am an only‐child and I don't know my father, so it has been the two of us. She had a period when we lived together with a lot of substance use and that was how I got in contact with people using substances. When I called her from treatment… I called and talked with her and I sent a letter to her about the fact that she was drinking too much—and then, all of the sudden, I was not welcome home anymore (laughs). So, to try to involve someone who have the same substance problems serves no purpose for your own recovery”.

W37: “When we get together, there is often conflict between mom, dad and me”.

W45: “I have a stepbrother. And, it is hard when he calls and says that he doesn't have a place to live. I say “Yes, but then you have to stay at X (shelter), because you can't come here”. I would have risked my own apartment, which I am very happy with and… I would most probably have risked using drugs, again, because I am very impulsive”.

There are few studies on family SOC and those few that exist have only found positive individual outcomes (Brodsky, 2009; Moscardino, Scrimin, Capello, & Altoè, 2010). The above examples, however, strongly suggest that the family can also be a source of negative SOC, of distinctiveness, abstention, frustration and alienage (see Mannarini et al., 2014). Maintaining positive relationships with family members who themselves use substances can be harmful to the process of recovery and are likely to manifest the process over time (Pettersen et al., 2018). In this situation, it is best to distance any person with substance use problems from substance using family members during a period after treatment. Again, it is likely that negative SOC and thereby a decision to keep in distance is likely to promote a process of recovery.

#### 5.4.2 Sober posttreatment communities

Sober posttreatment communities where generally addressed by the informants as positive for their recovery processes. However, in some cases, such communities (e.g., AA and low‐threshold services) could be harmful to the recovery process. As also underlined by the coresearcher, these cases most probably happen when the communities only become a reminder of a substance‐related identity or if there are groupings within the community that in fact promote exclusion of new members. Another informant also explained how such groupings within sober posttreatment communities, in fact, could hinder inclusion and recovery:

W42: “…what is a bit sad it that X (posttreatment community) is basically a very, very good offer. However, I have experienced that there is a grouping, which keeps to itself there, and… they may not be the most resourceful, but they are very resourceful when it comes to keeping people outside, to put it that way”.

I: “Ok, so there is exclusion? Not inclusion?”

W42: “I was very welcomed in the community in the beginning, to the point that they found out about my past. Then I was still very welcome among some, but there was of course someone who… absolutely did not feel that I belonged there, and who actually made life miserable and uncomfortable… like… it was hard to do anything about the situation directly and confront it… It's supposed to be a place for everyone but it isn't that”.

The peer researcher, here supplemented the above data mentioning that these groupings often take form “at the street” before treatment and are sustained in posttreatment communities. This finding illustrates the dark side to SOC: one person's strong SOC may be another person's exclusion.

### 5.5 Theme 5: Ideal communities

People develop their own meaning systems of ideal or positive communities (Bahl et al., 2017; Glynn, 1981; Heintzelman & King, 2014; Resnick & Leddy, 2015; Sagy et al., 2015). The informants in this study described their meaning systems of ideal communities in a way similar to positive relational communities; communities offering a broad range of meaningful activities, as a place where one can feel useful and learn from others who had a longer experience with recovery. One informant explained the value of using creative skills to master and recover from substance use problems as follows:

I: “Is there anything else that you want to say something about?”

M48: “Just that it could have been better. Ideally, there could have been more communities like, where you could meet, and play a little, and yes… make use of the experiences we have. Because there are a lot of experiences, or a lot of competence among substance users. There are many skilled people, who could have used their hands more”.

I: “So, a place where one could explore some activities; do different things, because that is something to build upon—doing things that you master?”

M48: “Mhm. I think that would have helped a lot, both for mastering substance use and things like that. At all”.

This finding suggest that persons with substance use problems have conceptualisations of ideal communities, which are clearly consistent with definitions of recovery: Feeling useful and accepted, doing meaningful activities with others, and developing skills and competence for recovery (Best & Laudet, 2010; Brekke et al., 2017; Moos, 2008). Moreover, the informants' descriptions of ideal communities suggest, contrary to agreements of a SOC and diversity as incompatible phenomena (Townley, Kloos, Green, & Franco, 2011) that a SOC and diversity can coexist in communities for substance use problems. As one female informant, participating in an exercise group explained:

W42: “It (the group) isn't specially for people with substance use problems. It is for people who are rehabilitating after surgery, maybe some who struggle with mental health issue and some who are very obese and who are troubled by that. It is a very nice mix of very different people. The fact that there isn't a focus on one particular group makes me feel included”.

People with substance use problems is a heterogeneous group and a group that is used to feel “different” from the majority. These daily life experiences of being different may be factors central for this tolerance and coexistence of diversity and SOC.

## 6 LIMITATIONS AND STRENGTHS

It is important to systematically consider the limitations and strengths of the study. First, none of the researchers was present in the interview setting. Therefore, valuable nonverbal information may have been missed. On the other hand, this procedure ensured that the researcher's theoretical perspectives and interests of the present study did not influence the original interviews. Second, the way the thematic analysis was carried out by the first author may be considered a limitation as it was focused only on identifying themes related to SOC. Other themes relevant for recovery may thereby have been overlooked. In a theory‐driven analysis, the data is forced into concepts included in the theoretical framework, thus influencing the coding of the data and thereby the findings of the study. However, a strength of the thematic analysis is the use of the peer researcher ensuring that SOC themes are being assessed from very different horizons. Third, this pilot study is based on the experiences of 16 informants from only four Norwegian municipalities, which restricts transferability to a more heterogeneous group and to the other 422 Norwegian municipalities. Follow‐up studies based on the present pilot have to be undertaken to investigate MSOC among specific groups of persons with substance use problems as well as larger samples from more of the Norwegian municipalities. Furthermore, this study captured the experiences of persons with substance use problems in a given time period and some of the experiences of the informants may have changed, as meaning systems are constantly changing. One of the strengths of this study, however, is as underlined that the two researchers—the first author and the peer researcher—represented very different meaning systems and everyday life experiences. By applying a collaborative research design, we have taken care of including the perspective of persons with substance use problems not only in the data but also in the coding and further analysis of the data. Future Norwegian clinical studies on substance use recovery should look into the utilities of additional collaborative praxis applied in other parts of the world, such as the inclusion of community members in research by using of community advisory boards. This is likely to increase the ecological validity of the research findings. Finally, in any community experience, there will be an interaction between individual characteristics, the community and the larger context. In this study, we have not focused on the role of personal characteristics or cultural context to fully understand these interactions.

## 7 CONCLUDING REMARKS

Currently, there is limited knowledge about the influence of different types of communities on recovery processes from problematic substance use. Most studies on SOC and substance use recovery have focused on the effect of therapeutic communities, or limited the study to one of the many communities of which people with substance use problems are members of. Our findings strongly suggest that experiences of both positive, as well as negative community connections within geographical, relational and ideal communities, take part in recovery processes. The findings, moreover suggest that recovery can be both assisted and natural and take place in communities within as well as outside the professional help system at the same time. Therefore, it is central to undertake more and larger MPSOC studies among people with substance use problems to get a more thorough understanding of the influence of communities on their recovery processes.

Regarding how different communities are experienced as influencing recovery, our findings confirm prior findings of key ingredients related to recovery: experiences of social bonding, belongingness, goal direction, meaning, but also having role models and social recovery capital, as well as building (recovery, social and creative) skills and competence, are important influences (Nelson & Prilleltenksy, 2010). However, our findings also illustrate new and additional aspects of the social situations of people with substance use problems. For example, for communities to promote recovery, the communities need to fulfil individual needs (e.g., age‐appropriate housing or meaningful activities), provide distance from pretreatment status, identity and roles and harmonize with individual meaning systems of an ideal community. Finally, our findings suggest that several communities, which are not usually thought of as communities (e.g., friends and different forms of posttreatment groups) are experienced as sources of SOC and recovery from problematic substance use.

On the basis of this pilot study we will already suggest that a community participation plan, based on the concept MPSOC, should be evaluated as a tool to be embedded in individual posttreatment follow‐up, outpatient treatment and in preventing potential destructive influence on recovery (e.g., the double burden of those who are deprived of help from their communities like the family, friends and neighbourhood, also being exposed to negative experiences when meeting with the professional help system within their municipality).

Future studies should look into the interactions between the therapeutic qualities multiple communities may have as means of natural recovery from problematic substance use and experiences with the professional help system when it comes to recovery processes (see Kogstad et al., 2013; Mayberry et al., 2009). In addition, we advise future studies to look into the relationship between MPSOC and concepts such as social recovery capital, hope and quality of life (see Best & Laudet, 2010; Stevens et al., 2018). Finally, it should be further investigated if SOC, as our findings suggest, is a bipolar construct for persons with substance use problems. Persons with substance use problems often have a large number of communities with negative SOC. These sources of negative SOC are often friends, family and the neighbourhood—the closest and most important relationships for development, attachment, care, support and resources. To promote recovery—assisted and natural—we, therefore, need a better understanding of how positive SOC(s) as well as negative SOC(s) can facilitate recovery throughout processes of recovery from substance use.

## ACKNOWLEDGEMENTS

The authors have no conflict of interest to disclose.

We would like to thank Marit Magnussen for her great efforts in the data collection. A special thanks to Kristin Tømmervik for valuable input on the manuscript. We also want to thank the Norwegian Directorate of Health for the assignment to study experiences of the services from Norwegian municipalities for persons with substance use problems.

## CONFLICT OF INTERESTS

The authors declare that there are no conflict of interests.

## Footnotes

1 The interviewer is represented with the code “I”.

2 Informants are represented with codes indicating their gender (F for female and M for male) and age. All quotes are translated by the first author.
